# *Mycobacterium ulcerans* Mouse Model Refinement for Pre-Clinical Profiling of Vaccine Candidates

**DOI:** 10.1371/journal.pone.0167059

**Published:** 2016-11-28

**Authors:** Angèle Bénard, Claudia Sala, Gerd Pluschke

**Affiliations:** 1 Swiss Tropical and Public Health Institute, Basel, Switzerland; 2 Global Health Institute, Ecole Polytechnique Fédérale de Lausanne, Lausanne, Switzerland; Fundació Institut d’Investigació en Ciències de la Salut Germans Trias i Pujol, Universitat Autònoma de Barcelona, SPAIN

## Abstract

Buruli Ulcer is a neglected tropical disease leading to extensive disabilities and morbidity in West Africa. In this paper we sought to characterize various strains of *Mycobacterium ulcerans* (*M*.*ulcerans*) with different origins and laboratory passage records while refining a mouse model for Buruli ulcer. We described, compared and followed the kinetics of the histo-pathological outcome of infection of a collection of strains at various anatomical sites of infection in order to find a suitable model for further immunization studies. Moreover we compared the outcome of infection in C57Bl/6 and Balbc/J mice. Specifically we described thoroughly one *M*. *ulcerans* strain characterized by slow growth rate and limited tissue necrosis, which presents close ressemblance with the infection kinetics in humans. This strain caused macrophages as well as T and B cells infiltration, correlating with mycobacterial proliferation at the site of infection as well as in the draining lymph nodes, making it a suitable strain to screen vaccine candidates efficacy.

## Introduction

Buruli ulcer (BU) represents the third most common mycobacterial disease after tuberculosis and leprosy. The disease has been reported in more than thirty tropical and subtropical countries, yet children in West-Africa carry the highest burden [1]. The disease often starts as a painless nodule, oedema or plaque, frequently located on the limbs of patients; it can nevertheless affect other body parts [1]. The non-ulcerative form of BU often progresses toward chronic ulcerative lesions showing characteristic undermined edges with destruction of the subcutaneous fat and skin tissue. Although some ulcers can resolve spontaneously, they often result in severe sequalae such as extensive scars, mobility limitations and in worst cases, amputations.

*Mycobacterium ulcerans (M*. *ulcerans)* is the causative agent for BU. It is closely related to *Mycobacterium marinum* known to infect fish and other aquatic animals [2]. Extensive analysis of the genome of *M*. *ulcerans* provided evidence for the existence of local clonal complexes in the BU endemic regions of Africa [3]. Genomic studies indicated that *M*. *ulcerans* emerged from a common ancestor with *M*. *marinum*, underwent extensive genome reduction and acquired a plasmid encoding a series of polyketide synthases involved in the synthesis of a macrolide toxin called mycolactone [4]. This toxin possesses immunosuppressive and cytotoxic properties and is a key virulence factor responsible for most of the pathogenesis of BU [5].

The transmission mode of *M*. *ulcerans* has not yet been clearly established [6] [1]. Human to human transmission does not seem to play a major role, but the occurrence of the disease is strongly associated with stagnant or slow flowing water bodies. Recent data also suggest the existence of genetic predisposition which could explain the observation that only a small proportion of exposed individuals seems to develop the disease [7]. *M*. *ulcerans* has been detected in aquatic biting insects and mosquitos. In a Southern Australian endemic area, two mammalian species, the common ringtail (*Pseudocheirus peregrinus*) and common brushtail (*Trichosurus vulpecula*) possums, have been identified as potential animal reservoirs for *M*. *ulcerans* and are thought to be the origin of transmission through mosquitos [8].

BU is a chronic necrotizing disease with delayed onset; a study in Southern Australia estimated a mean incubation period of 4.5 months (34–264 days). Until recently, wide surgical excision of lesions was the only treatment option for BU. Since 2004, WHO recommends a combination drug treatment with rifampicin and streptomycin for eight weeks requiring long hospital stays (Buruli ulcer, *Mycobacterium ulcerans* infection, WHO Fact sheet N°199 August 2012). The morbidity associated with BU in endemic area could be prevented by a vaccine against *M*. *ulcerans* infections. However, it is not clear whether cellular or humoral immune responses are primarily required to contain the infection. Mycolactone has been shown to induce necrosis and apoptosis in the tissue surrounding the established infection foci. As a result infiltrating cells of the innate and adaptive immune system usually do not reach the clusters of extracellular mycobacteria found in advanced lesions. However, memory cells elicited by a vaccine may enable the immune system to eliminate the infection in an early stage when mycolactone levels are still low. In order to profile candidate vaccines, we sought to develop a mouse model that allows us to follow the course of an experimental *M*. *ulcerans* infection. Several studies have been performed using *M*. *ulcerans* infection in mice [9–11]. These studies differ in several parameters, including *M*. *ulcerans* strains, inoculation dose, site of inoculation and mouse strains. Here we have systematically compared these parameters with the aim of establishing a reliable and reproducible mouse model that could be exploited for future immunization studies.

There is evidence that *M*. *ulcerans* behaves as an intracellular pathogen within the first phases of infection before lysing the host macrophage and becoming extracellular within the surrounding necrotic fibrotic area [12]. We therefore sought to investigate the influence of the genetic background of infected mice on the development of *M*. *ulcerans* infection. In the majority of studies of *M*. *ulcerans* infection, BALB/cJ mice, which display a Th2 polarized immune response, were the model of choice [10][11][9]. Only a few studies describe *M*. *ulcerans* infection in C57Bl/6 mice, whose immune response is characterized by a Th1 polarization [13][14]; moreover these mice are commonly used in *M*. *tuberculosis* infection models [15]. Our results indicate that this difference has only minor impact on the outcome of an *M*. *ulcerans* infection.

## Materials and Methods

### *M*. *ulcerans* culture

The *M*. *ulcerans* strains NM20/02, NM05/02, NM13/02, NM15/02, NM18/02 were originally isolated from Ghanaian BU patients as described in Yeboah-Manu et al and were kindly provided by the Institute for Infectious Diseases in Bern [16,17]. The strains *M*. *ulcerans* S1012 and S1013 were isolated from the ulcerative lesion of a Cameroonian BU patients in 2010[1]. Stocks were stored at -80°C. BacTAlert bottles (Biomerieux diagnostic—France) were inoculated and cultures were grown at 30°C for 3 weeks and passaged once before preparing the inoculum.

For animal inoculation the bacterial suspensions were centrifuged at room temperature for 20 minutes at 1800g. The supernatant was discarded and the pellet was weighed and resuspended in sterile phosphate buffered saline (PBS), pH 7.4. Ten-fold dilution series of this suspension were prepared and selected doses were used as inoculum. Serially diluted suspensions were plated on Middlebrook selective 7H11 plates (Becton-Dickinson, Sparks, MD). Plates were incubated at 32°C and colonies were counted after 8 weeks with a final determination at 10 weeks of incubation. While strain NM20/02 has been extensively sub-cultured, all other strains had only been subcultured a few times (two to five) before use.

### Ethical statement

All the mouse experiments were approved by the Ethics and Veterinary office regulations of the state of Vaud (SAV), Switzerland and documented in the protocol of administrative authorization number 2261. Infection experiments with *M*. *ulcerans* were conducted under Biosafety-level-3 conditions at the École Polytechnique fédérale de Lausanne (EPFL). Ethical clearance for the isolation of the *M*. *ulcerans* strains NM20/02, NM05/02, NM13/02, NM15/02, NM18/02 was obtained from the institutional review board of the Noguchi Memorial Institute for Medical Research, NMIMR, with the federal wide assurance number FWA00001824. Ethical clearance and aproval of the study including the isolation of S1013 and S1012 were obtained from the Cameroon National Ethics Committee (N°041/CNE/DNM/09 and N°172/CNE/SE/2011) and the Ethics Committee of Basel (EKBB, reference no. 53/11).

### Mice

Groups of 5 mice either C57/Bl6 (Charles River) or BALB/cJ (Janvier), were inoculated subcutaneously with 30microliters of bacterial suspension in the left footpad, in the left hock or in the left ear. We evaluated the hock as a new site of infection specifically to minimize the suffering of the animal compared to footpad injection as described in Kamala [18]. Mice were monitored and documented every two weeks for development of lesion. At 5, 10 and 15 weeks after inoculation, mice were sacrificed using CO_2_ inhalation and the injected tissue harvested aseptically in 10% neutral-buffered formalin solution (approx. 4% formaldehyde, Sigma). After 24 hours, tissue samples were washed with ethanol and stored at 4°C until further processing. Footpads, ear and hocks were then incubated in decalcification solution, consisting of 0.6M EDTA (Ethylenediaminetetraacetic acid) and 0.25M citric acid, for 10 days at 37°C under shaking conditions. After decalcification of bones, tissues were embedded in paraffin, cut into 5μm sections using a microtome, and retrieved on glass slides.

### Histology and immunohistochemistry

For histopathology, foot pad, hock and ear 5-μm thin sections were deparaffinised, rehydrated, and stained with Haematoxylin/Eosin (HE, Sigma, J.T. Baker) or Ziehl-Neelsen/Methylene blue (ZN, Sigma) according to WHO standard protocols. Stained sections were mounted with Eukitt mounting medium (Fluka). Pictures were taken with a Leica DM2500B microscope or with an Aperio scanner.

For immunohistochemistry, sections were deparaffinized and rehydrated. Endogenous peroxidase was blocked with 3% H_2_O_2_ for 10 min and unspecific binding was prevented by incubation with blocking serum matching the secondary antibody host. Subsequently, slides were pretreated by the hot-borate antigen retrieval method (0.02 M, pH 7) [19] and incubated at room temperature with monoclonal antibodies against CD45R (B cells; clone RA3-6B2) (Serotec), monocytes/macrophages (Mo-Ma; clone MOMA-2) (Serotec), CD3 cells (T cells; clone CD3-12) (Serotec), neutrophils (clone NIMP-R14) (Abcam). Afterwards, sections were incubated for 30 min with a corresponding biotinylated secondary antibody (Vector Laboratories) and for an additional 30 min with streptavidin-horseradish peroxidase conjugate (Vectastain ABC kit; Vector Laboratories). Staining was performed using Vector NovaRed (Vector Laboratories) and Meyer's hematoxylin as a counterstain (Sigma). Sections were mounted with Eukitt mounting medium (Fluka). Pictures were taken with a Leica DM5000B microscope. Histo-pathological photographic pictures displayed are representative of results obtained for each experiments.

## Results

### Various degrees of virulence of *M*. *ulcerans* strains

In order to establish an experimental *M*. *ulcerans* mouse infection model for the evaluation of the protective efficacy of candidate vaccine formulations, we compared the virulence of seven different clinical *M*. *ulcerans* isolates from Ghana and Cameroon. While one strain (NM20/02) had been recurrently sub-cultured *in vitro*, others had either been passaged only a few times (NM05/02, NM13/02, NM15/02, NM18/02), namely less than five times, or cryopresevered after only one or two subculturing steps following isolation from the lesions of BU patients (S1012, S1013) (Table 1).

**Table 1:** Origin and subculture history of *M*.*ulcerans* strains.

Strain	Country of origin	Extent of subculture
NM20/02	Ghana	extensive
NM13/02	Ghana	moderate
NM05/02	Ghana	moderate
NM15/02	Ghana	moderate
NM18/02	Ghana	moderate
S1013	Cameroon	low
S1012	Cameroon	low

**Fig 1:**
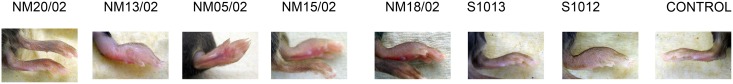
Comparison of macroscopic signs of infection in mice inoculated by various *M*. *ulcerans* strains. Suspensions of *M*. *ulcerans* from seven strains from Ghana and Cameroon were injected subcutaneous (*s*.*c*.) in the footpad of C57Bl/6 mice. Footpad swelling at week 4 after infection is shown.

We hypothesized that the amount of extra-cellular matrix injected alongside the bacteria could influence the outcome of infection and the degree of inflammation. Hence we chose to standardize our inoculum according to wet mass while determining a posteriori the amount of CFU contained within the innocula. Strains grown in BacT Alert bottles were centrifuged, resuspended in PBS and between 2.4x10^4^ and 8x10^5^ colony forming unit (CFU) were injected in a volume of 0.03ml subcutaneously in the left footpad of C57/Bl6 mice (S1 Fig). Footpads of mice were monitored every two weeks for the development of swelling, redness and ulceration. The first signs of footpad swelling appeared between two and five weeks after inoculation in all mice. Ulceration never occurred in NM20/02 infected mice while perfusion and ultimately open lesions arose between two to four weeks after the first signs of swelling in 5 out of 5 mice of each group inoculated with the low-passaged strains, namely NM05/02, NM13/02, NM15/02, NM18/02. As shown in Fig 1, noticeable differences were seen in the ability of each strain to induce inflammation. In contrast to all other isolates, the extensively cultivated strain NM20/02 persisted for prolonged periods of time in the footpads without causing massive pathology. This strain showed the highest growth rate of all isolates *in vitro* and had retained the capacity to produce mycolactone (S1 Fig and data not shown). While a mild swelling of the footpad became apparent four to five weeks after injection of 8x10^5^ CFU of NM20/02, none of the inoculated mice injected with this strain developed an ulcerative lesion. This attenuated phenotype did not lead to massive tissue destruction but evolved slowly and mirrored in this respect more closely the progressive evolution of human BU lesions, NM20/02 may represent a particularly suitable indicator strain for vaccine efficacy testing. Hence we characterized the progression of the infection provoked by NM20/02 in further detail.

**Fig 2:**
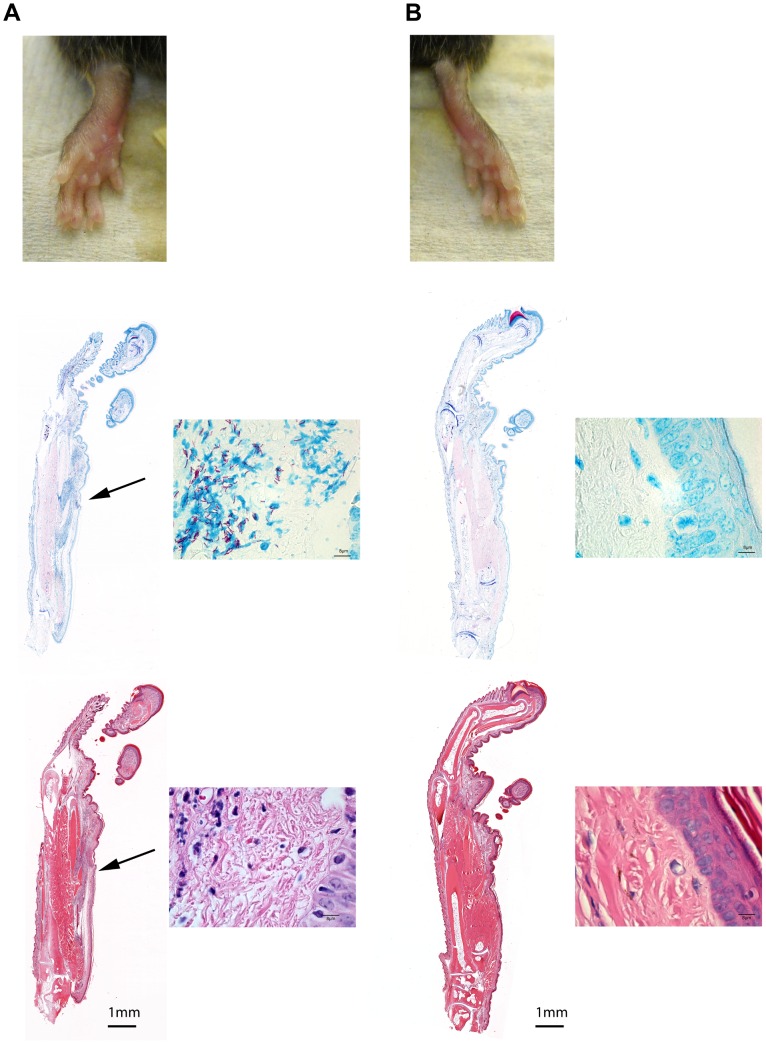
Macroscopic and microscopic signs of infection and inflammation in mice infected with *M*. *ulcerans*. The upper panel shows photograph of a C57Bl/6 mouse footpad 5 weeks after injection with *M*. *ulcerans* (NM20/02) (A) and the non-injected control footpad (B). Middle panel shows the corresponding 5micrometer thick paraffin embedded footpad slices stained with Ziehl-Neelsen (ZN) staining. Finally, the lower panel shows the same footpad slices stained with Hematoxylin Eosin (H&E). Arrows show the bacterial foci (ZN) and the inflamed tissue (H&E) of the lesion.

**Fig 3:**
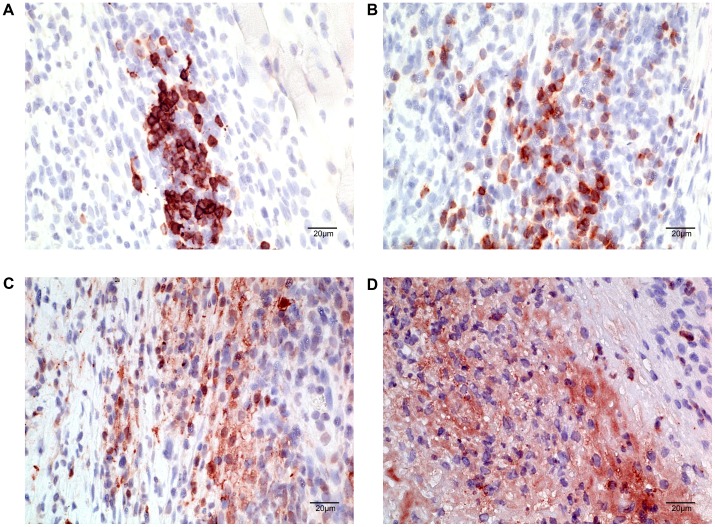
Infiltration of leukocytes within the footpad infected with *M*. *ulcerans*. Immunohistochemistry was performed on 5 micrometer paraffin embedded sections of footpad of mice infected with a suspension of *M*. *ulcerans* (NM20/02) in order to identify the nature of the leukocytic infiltrate present within and around the bacterial foci. B cells were stained with anti-CD45R antibody (A), T cells with anti-CD3 antibody (B), Macrophages with MOMA (C) and neutrophils with anti-Ly6G/Ly6C (D).

ZN staining of thin sections of the footpads five weeks after inoculation revealed solid-stained acid-fast bacilli (AFB) located in the subcutis of the skin tissue close to the site of injection (Fig 2). AFB were surrounded by infiltrates composed of numerous polymorphonuclear (PMN) leukocytes and located in close association with irregularly shaped blue stained nuclei. Compared to the control footpad, hematoxylin-eosin (H&E) staining of the infected footpads revealed signs of extensive dermal edema as well as coagulative necrosis around the bacterial foci. The infiltrated area was loosely organized in a central necrotic core mainly composed of neutrophilic debris with granular cells characterized by vacuolar degeneration and seen in close proximity to the bacterial foci (Fig 3C and 3D). Macrophages formed a loose and irregular network around this neutrophilic necrotic core, while T and B cells more distantly located from the bacteria looked intact with a well-defined cytoplasm and a round shaped nucleus (Fig 3A and 3B). B cells were often seen in clusters but typical organized granuloma structure was not observed [20].

### Kinetics of *M*. *ulcerans* infection in mice

**Fig 4:**
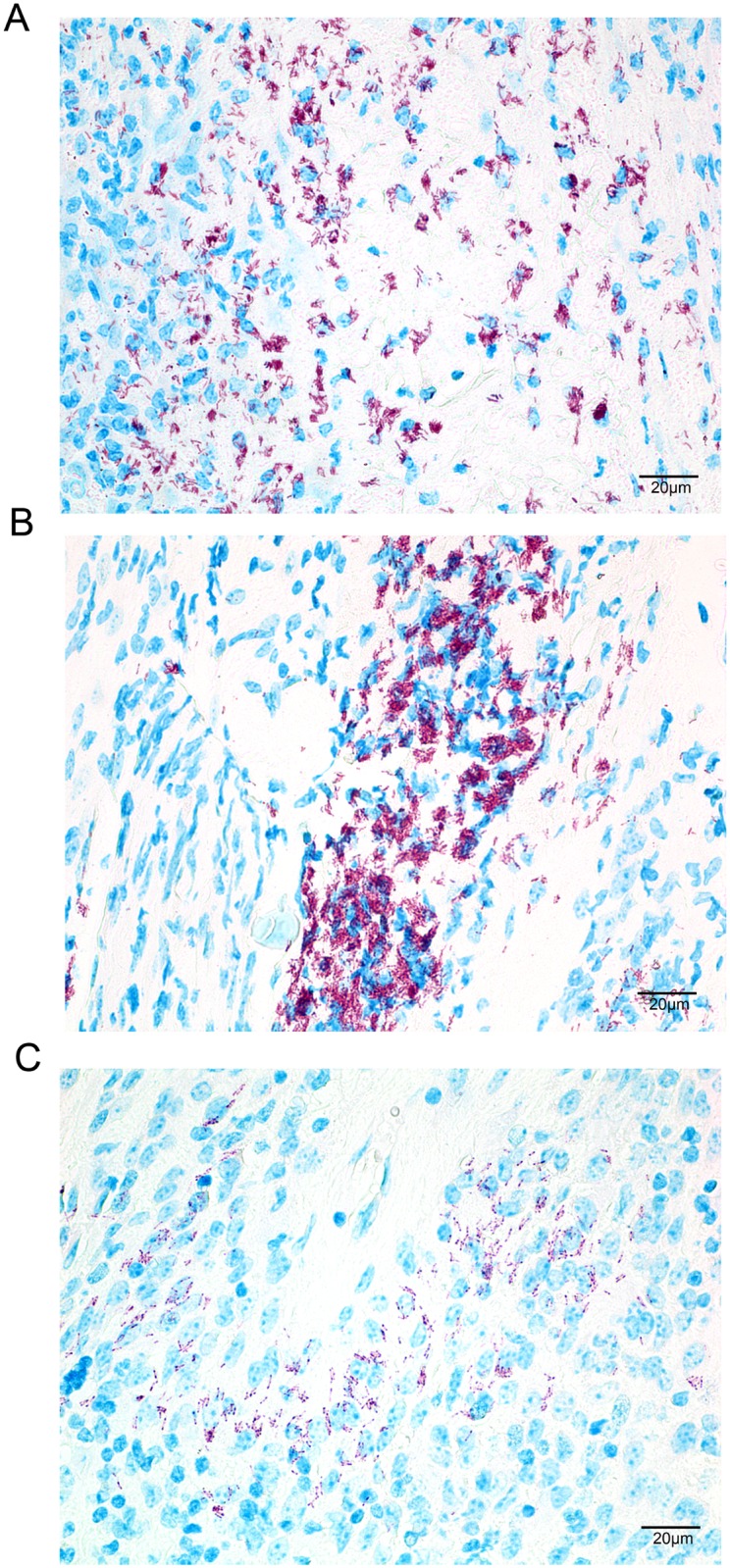
Evolution of bacterial load over time after infection by *M*.*ulcerans*. ZN stainings of 5 micrometer thick slices of paraffin embedded footpad of a mouse infected *s*.*c*. with *M*. *ulcerans* NM20/02 4 weeks (upper), 10 weeks (middle) and 15 weeks (lower) after infection.

**Fig 5:**
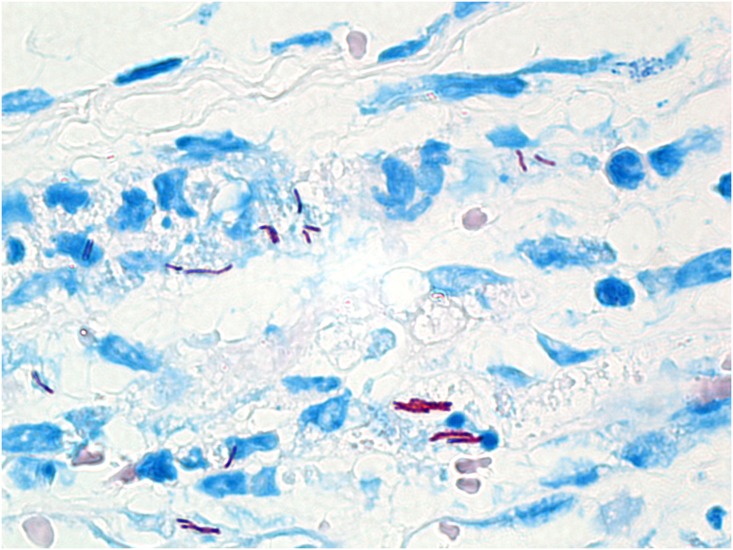
Long-term persistence of *M*.*ulcerans* bacteria after infection. ZN staining of 5 micrometer thick paraffin embedded sections of mice injected subcutaneously in the ear with *M*. *ulcerans* NM20/02. Tissue samples were harvested 7.5 months after infection.

To assess whether strain NM20/02 can multiply and persist for an extended period of time within the host, we followed the fate of inocula injected into the footpad in C57/Bl6 mice over time. In order to monitor the development of the infection after *M*. *ulcerans* NM20/02 inoculation, we designed a time course experiment in which we assessed bacterial growth by histological staining at various time points of the experiment. After injection, Ziehl-Neelsen (ZN) staining of sections from the site of injection, i.e. footpad, demonstrated clear multiplication of the bacteria, increasing from week 4 to week 10 and assembling progressively into bacterial foci (Fig 4A and 4B). However beaded bacteria were observed 15 weeks after inoculation showing heterochromatic nuclei of PMN leukocytes surrounding dying bacteria (Fig 4C). These observations may suggest a halt in bacterial burden and a decrease in bacterial viability. While leukocytic infiltration was extremely abundant at week 5, it seemed to decrease by week 10 but persisted until week 15, the last time point evaluated. On the other hand, a follow-up experiment where 2.8x10^5^ CFU/mL of *M*. *ulcerans* NM20/02 was innoculated in the ear of BALB/cJ mice showed that solid stained AFB and sustained infiltration could still be found 7.5 months after injection (Fig 5). While some areas clearly showed beaded bacteria indicative of poor viability, it revealed that mycobacterial antigens are able to persist and to maintain sustained inflammation for several months after inoculation.

**Fig 6:**
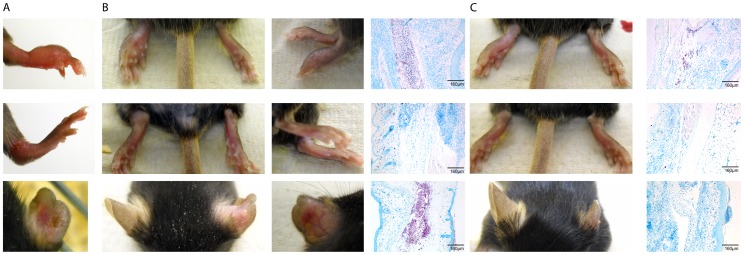
Evaluation of various sites of infection for *M*.*ulcerans* infection. A solution of *M*. *ulcerans NM20/02* was injected *s*.*c*. in three different sites of C57Bl/6 mice, namely footpad (upper panel), hock (middle panel) and ear (lower panel). The following time points after infection are displayed: week 2 (A), week 4 (lower panel—B) or 5 (upper and middle panel—B) and week 10 (C). The corresponding ZN staining of 5 micrometer thick slices of paraffin embedded footpad, hock and ear at week 4 (B) and week 10 (C) after infection are shown.

In order to assess the optimal site of injection for *M*. *ulcerans* in the mouse, we compared the outcome after injection of strain NM20/02 in the hock, footpad and ear of C57/Bl6 mice. Development of lesions at the hock are thought to obstruct movement of animals less than lesions at the footpad [18]. As shown in Fig 6, injection in the footpad and in the ear readily induced a macroscopically detectable inflammation that was not seen in the hock. Furthermore injection in the hock proved unpractical, since the bacterial foci were difficult to localize during histo-pathological analyses (Fig 6 middle panel). Injection of the ear is technically demanding and resulted in rapid loss of tissue (Fig 6 lower panel). For routine use, the footpad met all the criteria for a safe and reproducible method of injection, inducing a macroscopically observable and microscopically measurable infection.

**Fig 7:**
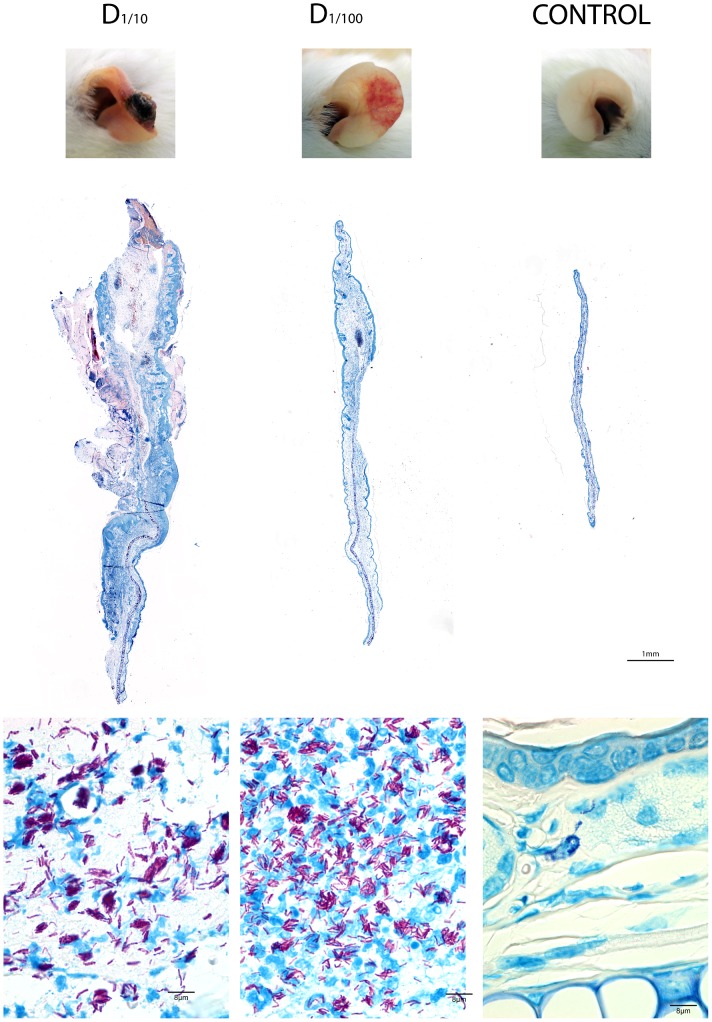
Dose effect of *M*.*ulcerans* inoculum on the outcome of infection. Photographs of ear (upper panel) and the corresponding ZN staining of 5μm thick slices of paraffin embedded ear (lower panel) of mice infected with various dilutions (D = 1/10 and D = 1/100) of *M*. *ulcerans* NM20/02 suspension 9 weeks after infection.

To assess the effect of infection dose, mice were injected with a serial 10-fold dilution of an NM20/02 suspension at the three different sites of injection. We have chosen to display here the most representative histopathology analysis pictures, namely the ear of BALB/cJ mice, as this site provided the most visually assessable macroscopic changes following the infection, illustrating best the dose effect. A similar dose response experiment was performed in the footpad and hock of both strains of mice and provided similar findings (S2 Fig). While the highest dose resulted in rapid and fulminating swelling with loss of ear tissue and ultimately development of a scar (data not shown), a 10x diluted inoculum resulted in a severe wound but no loss of tissue (Fig 7 left panel). Finally a 100x dilution dose only induced limited swelling of the ear and signs of redness (Fig 7 middle panel) compared to the negative control showing no sign of inflammation (Fig 7 right panel). At the microscopic level, marked differences in leukocytic infiltration, edema formation and necrotic area reflected the macroscopic outcome and the number of ZN positive bacilli as well as their dissemination within the tissue. It is noticeable that the highest dose injected resulted in tissue discharge and subsequent wound closure leaving healthy and less affected tissue with scarce bacilli. It is not clear whether the rare bacilli detected within the healthy tissue were viable and could potentially reactivate the infection. Similar findings were observed at the other injection sites (cf. S2 Fig).

**Fig 8:**
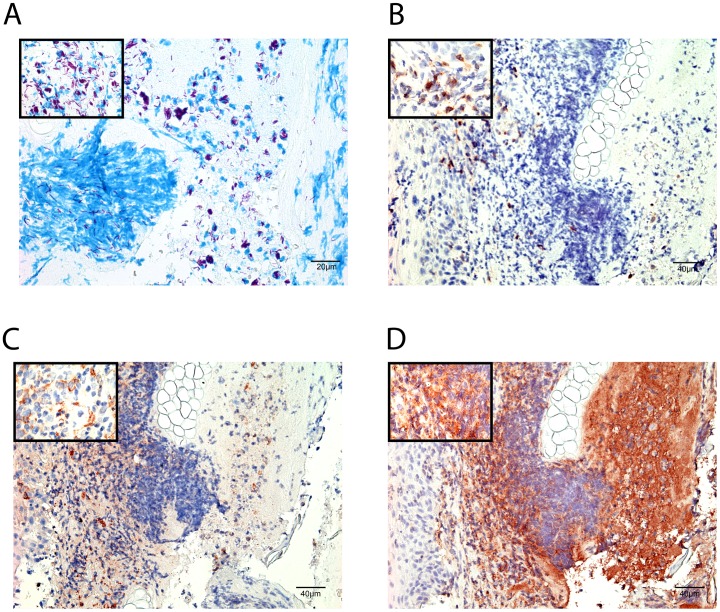
Infiltration of leukocytes within the ear infected with *M*. *ulcerans*. ZN staining (A) and Immunohistochemistry (B-D) were performed on 5 micrometer paraffin embedded ear sections of Balb/c mice sacrificed at week 9 after infection with a suspension of bacteria diluted at D_1/10_ in order to identify the nature of the leukocytic infiltrate present within and around the bacterial foci. T cells were stained with anti-CD3 antibody (B), macrophages with MOMA (C) and neutrophils with anti-Ly6G/Ly6C (D).

In the tissue of BALB/cJ mice inoculated with lower doses of NM20/02 (D_1/10_ –right), numerous AFB were found loosely associated with necrotic nuclei. On the other hand, in mice receiving the D_1/100_ dilution dose, AFB were primarily located nearby polymorphonuclear leukocytes. Immunohistochemistry analysis revealed a large necrotic area composed of neutrophilic debris in close proximity with the bacilli (Fig 8D). T cells and macrophages on the other hand were located in the periphery of the bacterial foci (Fig 8B and 8C).

### Outcome of *M*. *ulcerans* infection in different mouse strains

**Fig 9:**
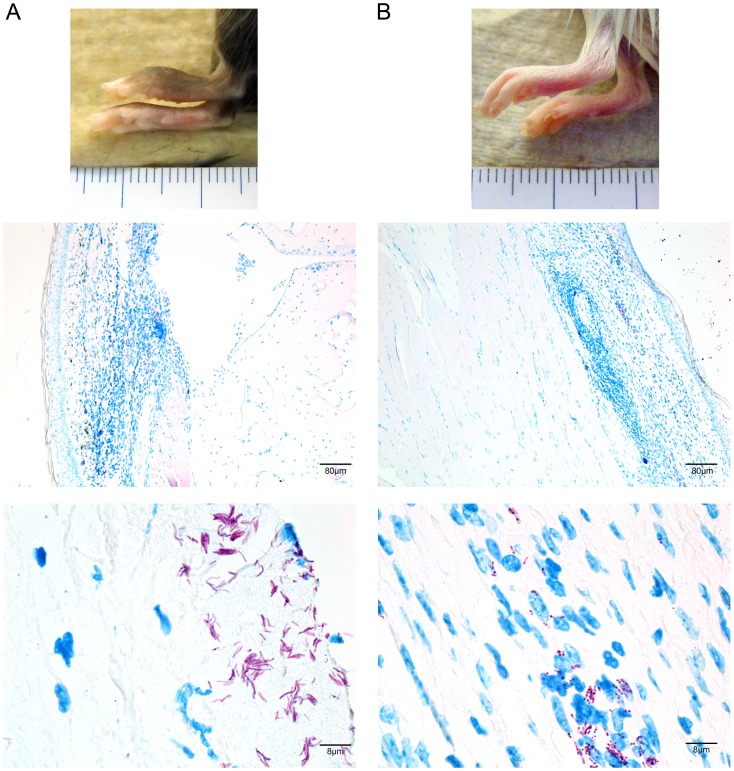
Influence of mouse strain on the outcome of *M*.*ulcerans* infection. A solution of 6.6x10^4^ CFU of *M*. *ulcerans* was injected in the footpad of C57Bl/6 (A) and Balb/cJ mice (B). Footpad pictures are shown at week 8 after infection (upper panel). ZN staining of 5μm thick histology slices of paraffin embedded footpads show infiltration area (blue nuclei—middle panel) and bacterial load (pink rod-shape bacilli—lower panel) 12 week after infection in C57Bl/6 (A) and Balb/c (B) mice.

In an attempt to examine the influence of a primarily Th2 versus Th1 oriented immune responses, we compared the development of the infection in BALB/cJ and C57Bl/6 mice after inoculation of footpads with 6.6x10^4^ CFU of strain NM20/02. BALB/cJ mice showed less swelling and less infiltration than C57Bl/6 mice at early time points (Fig 9). Moreover bacterial multiplication in BALB/cJ mice was slightly lower than in C57Bl/6 mice (Fig 9). Furthermore the proportion of solid stained AFB was higher in C57Bl/6 mice than in BALB/cJ mice (Fig 9).

**Fig 10:**
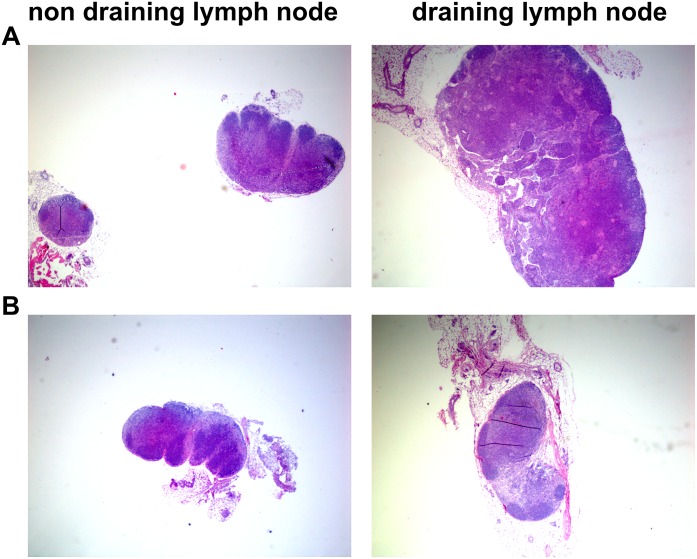
Lymph node cellularity of Balb/Jc vs C57Bl/6 mice. H&E staining was performed on 5 micrometer paraffin embedded sections of draining (right) and non-draining lymph nodes.

Finally a clear difference in lymph node cellularity was observed between BALB/cJ and C57Bl/6 mice as illustrated in Fig 10 where draining lymph nodes from infected C57/Bl6 mice were markedly more enlarged than draining lymph nodes from infected BALB/cJ mice. Furthermore an increased number and size of germinal centers in the draining lymph nodes of C57Bl/6 mice compared to the draining lymph nodes of BALB/cJ mice suggested a more vigorous immune response in the C57Bl/6 mice (Fig 10 and data not shown). Western blotting analyses and ELISAs with bacterial lysates as well as recombinant *M*. *ulcerans* proteins revealed in none of the two mouse strains the development of a significant *M*. *ulcerans* specific antibody titer even after 15 weeks of infection (data not shown).

## Conclusion and Discussion

The overall goal of this study was to describe features of a chronic *M*. *ulcerans* mouse infection model suitable for the profiling of vaccine candidates.

In our study it is noteworthy that the quantification of bacteria injected was based on wet biomass weight evaluation and subsequent CFUs determination, which does not always allow precise quantification of live single *M*. *ulcerans* bacteria in the inoculum. The structure of the mycobacterial cell wall in addition to the presence of extracellular matrix and the resulting tendency to aggregate in clumps complicates the accurate measurement by classical methods commonly used in microbiology such as optical density measurements. We sought to minimize this factor by using the exact same conditions of culture and preparation of the bacteria inoculated in each experiment and to normalize inoculates by the weight of their wet mass prior to injection. While automated image analysis for quantification of bacterial cells from digital microscope images, RNA isolation of the tissue at the site of infection followed by reverse transcription qPCR and eventually CFU determination from tissue harvested at the site of infection and at various time points would help determining the viability and quantification of the bacteria at the endpoint of infection, they were beyond the capacity and scope of this study.

Based on a thorough histological analysis of several parameters at different time points in the time course of the infection for each of the strains available in this study, we were able to identify the most suitable strain for further immunization studies. Infection with strain NM20/02 resulted in the development of edematous lesions with widespread lymphocytic infiltration, multiplication and persistence of the bacteria for several weeks without leading to open ulceration and tissue loss. Hence strain NM20/02 caused mild infection and massive local inflammation shown by immune cells infiltration, which should make it possible to characterize mechanisms of immune protection elicited by vaccination. On the other hand, strains characterized by low passage numbers *in vitro* display a higher virulence inducing greater inflammation and rapid necrosis in the host tissue precluding analysis of immunological process at the histological level. These differences could not be attributed to mycolactone production since all strains produced similar amounts of mycolactone (data not shown).

Our results confirmed that the footpad is the site of choice in the mouse for a well-defined, consistent and reproducible infection since it is most suitable to monitor both infiltration and multiplication of the bacteria by histo-pathological analysis.

By comparing the infection outcomes in two different mouse genetic backgrounds, we showed that C57Bl/6 mice display more extensive leukocytic infiltration and bacterial growth than BALB/cJ mice. The size and cellularity of the draining lymph nodes in C57Bl/6 mice were markedly higher, indicative of a predominant cellular immune response. While polarization towards a Th1 response is thought to play a preponderant role in fighting against mycobacterial infections, its role may be more complex in *M*. *ulcerans* infection and requires further elucidations. Eventually passive immunization with various antibodies directed against the 18kDa heat shock protein (MUL2232) and the 27kD laminar binding protein (MUL3720) either in BALB/cJ or in C57Bl/6 mice failed to protect mice against *M*. *ulcerans* infection (data not shown).

## Supporting Information

S1 Fig CFUs from innoculum of *M*.*ulcerans* strains. A solution of *M*.*ulcerans* from 6 different strains of various geographical origins was injected in the footpad of C57Bl/6 mice. CFUs are represented as CFU/ml of innocula for each strain at the time of injection.
(TIF)

S2 Fig Dose response of NM20/02 in the footpad. C57Bl/6 mice were injected with various dilutions (D = 1/10 and D = 1/100) of *M*. *ulcerans* NM20/02 suspension. Photographs of the footpad at week 2 after infection (left panel) and ZN staining of 5μm thick slices of paraffin embedded tissue 5 weeks after infection (right panel, magnification x100 and x400).
(TIF)
