# Supplementary figures and images for: Mycobacterium ulcerans Mouse Model Refinement for Pre-Clinical Profiling of Vaccine Candidates
Source: PLoS One. 2016 Nov 28;11(11):e0167059. doi: 10.1371/journal.pone.0167059 (PMC5125663; doi:10.1371/journal.pone.0167059)

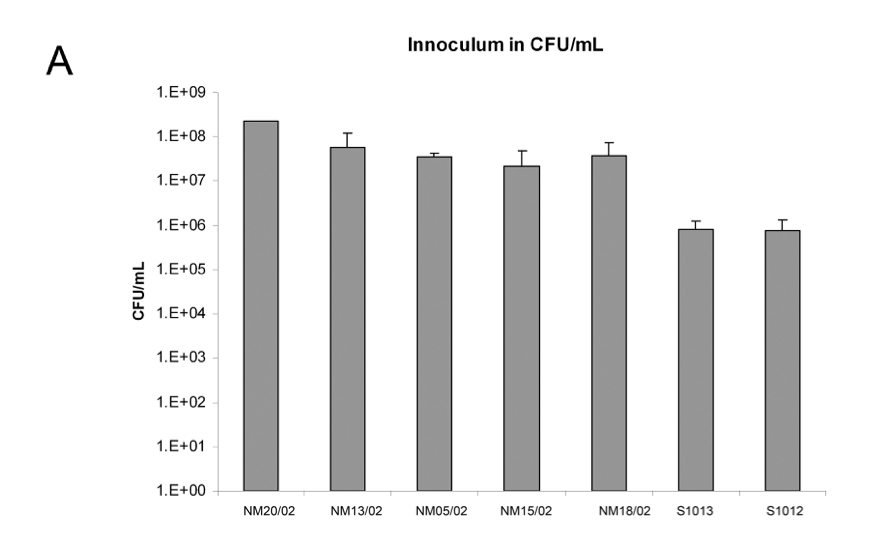

Supplement: S1 Fig — A solution of M.ulcerans from 6 different strains of various geographical origins was injected in the footpad of C57Bl/6 mice. CFUs are represented as CFU/ml of innocula for each strain at the time of injection. (TIF) [file pone.0167059.s001.tif]

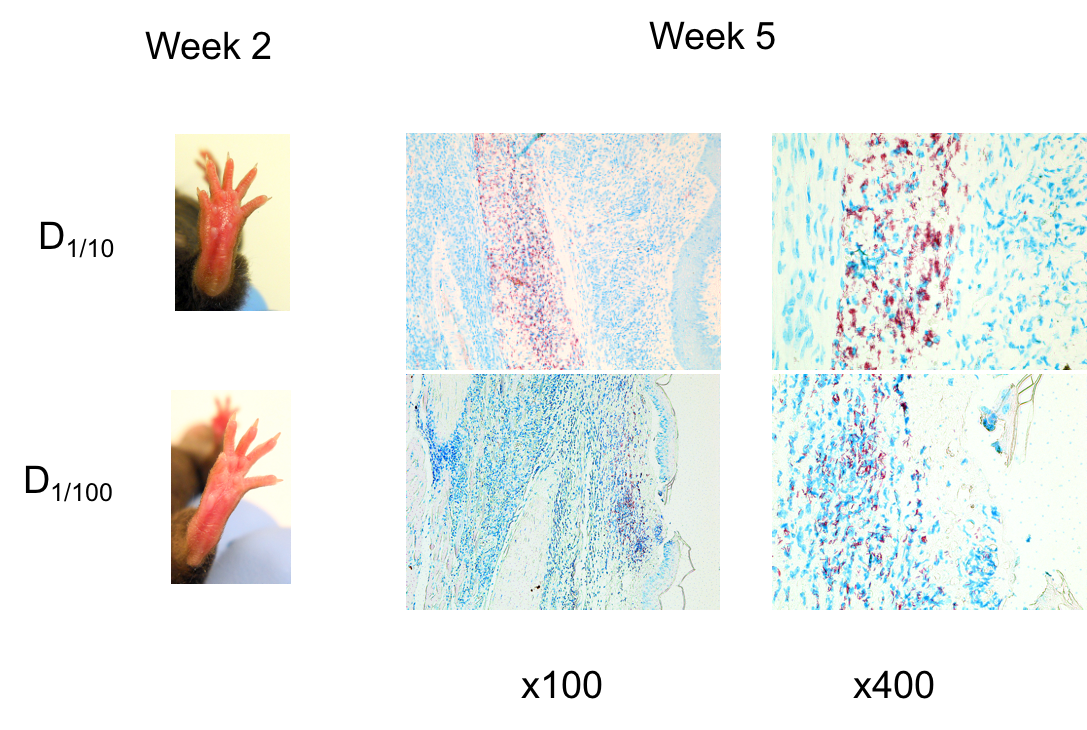

Supplement: S2 Fig — C57Bl/6 mice were injected with various dilutions (D = 1/10 and D = 1/100) of M. ulcerans NM20/02 suspension. Photographs of the footpad at week 2 after infection (left panel) and ZN staining of 5μm thick slices of paraffin embedded tissue 5 weeks after infection (right panel, magnification x100 and x400). (TIF) [file pone.0167059.s002.tif]
